# Modelling the thermal behaviour of a building facade using deep learning

**DOI:** 10.1371/journal.pone.0207616

**Published:** 2018-12-21

**Authors:** Fidel Aznar, Victor Echarri, Carlos Rizo, Ramón Rizo

**Affiliations:** 1 Department of Computer Science and Artificial Intelligence, University of Alicante, San Vicente del Raspeig, Alicante, Spain; 2 Department of Architectural Constructions. University of Alicante, San Vicente del Raspeig, Alicante, Spain; Pavol Jozef Safarik University in Kosice, SLOVAKIA

## Abstract

This article aims to model the thermal behaviour of a wall using deep learning techniques. The Fourier theoretical model which is traditionally used to model such enclosures is not capable of considering several factors that affect a prediction that is often incorrect. These results motivate us to try to obtain a better thermal model of the enclosure. For this reason, a connexionist model is provided capable of modelling the behaviour of the enclosure from actual observed temperature data. For the training of this model, several measurements have been obtained over the course of more than one year in a specific enclosure, distributing the readings among the different layers of it. In this work, the predictions of both the theoretical model and the connexionist model have been tested, contrasting them with the measurements obtained previously. It has been observed that the connexionist model substantially improves the theoretical predictions of the Fourier method, thus allowing better approximations to be made regarding the real energy consumption of the building and, in general, the prediction of the energy behaviour of the enclosure.

## 1 Introduction

The thermal behaviour of the walls that make up the facades of buildings is undoubtedly one of the most important research fields in the search for a reduction in annual energy demand [1–3]. The transfer of heat between the indoor and outdoor environment facilitated by these construction elements depends, to a large extent, on some physical parameters of the materials that make them up [4], on their permeability to the passage of air or steam, on the quality of the construction or on the incidence of thermal bridges generated by the discontinuity of the thermal insulation materials. This transfer of heat and air infiltration affects the hygrothermal conditions of the indoor environment [5–7], and affects energy consumption through HVAC systems to achieve and maintain the comfort levels demanded by users and regulated by regulations [8, 9].

The Fourier law, valid for parallel layers of materials with unlimited surface area, a stationary indoor air temperature regime *Ti* and outdoor *Te*, establishes the temperature gradient that multi-layer facades acquire when there is a thermal jump of temperatures between the outdoor air and the indoor air, as shown in Eq 1. However, this law is only valid for ideal situations [10]. The walls have limited dimensions, discontinuities due to carpentry and glazing, thermal bridges due to construction requirements [11], some permeability to the passage of air, or risk of interstitial condensation.

There are other methods to obtain highly accurate results on the determination of optimum insulation thickness, while some authors used a numerical method based on the implicit finite volume procedure under steady periodic conditions [12–17], the others used an analytical method based on Complex Finite Fourier Transform [18, 19]. Generally, the results and conclusions from these studies are site specific and applicable only to local climatic conditions, walls have limited dimensions and present discontinuities due to this metalwork, woodwork, glazing. Thermal bridges also exist due to construction requirements, discontinuities etc. [20, 21]. Moreover, when the time dependence of internal temperature and energy consumption of a whole building must be evaluated, that is more complex and requires greater computing capacity, computer resources and time [22].

For these reasons, we propose a system capable of predicting the behaviour of this facade in a more reliable way. There are similar works in this line, using neural networks for the prediction of thermal transmittance of windows [23], heat transfer coefficients [24] and moist porous materials [25]. These studies do not take into account temporal sequences, since they are only aimed at obtaining coefficients.

For this work we require a set of temperature measurements, similar to those obtained in [26, 27]. A series of real temperature data is available, obtained by monitoring a facade of a residential building located in Alicante, Spain, on the Mediterranean coast. This facade was monitored for more than one and a half years from January 2012, in order to be able to properly evaluate its thermal behaviour in the different stations, and to analyse in a comparative way the effect of the thermal inertia of its materials, and incident solar radiation.

This research focuses on the development of a deep learning system capable of predicting the thermal behaviour of the facade. The development in recent years of deep learning techniques for the modelling of all types of systems, including image analysis, the development of voice analysis systems, and the prediction of time series [28–30], demonstrates its great potential to extract models from a sufficient set of data.

This article will initially characterise the facade to be modelled. Next, the theoretical Fourier model will be presented in detail, analysing its operation compared to the actual data acquired. Subsequently, a deep connexionist model will be provided to learn the characteristics of the facade. The functioning of the model presented will be evaluated, and a discussion regarding the proper functioning of the deep model presented with respect to the Fourier theoretical model will conclude the article.

## 2 Enclosure description

**Fig 1:**
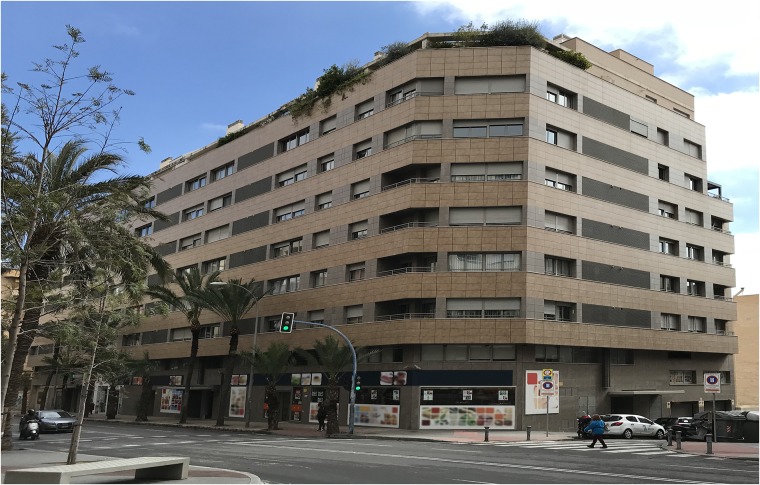
View of the corner between Avenida Benito Prez Galdós and Catedrático Ferre Vidiella Street (north and west facade), where the building being studied is located.

The building under study is located in the city centre of Alicante (Fig 1). It is a corner building whose facades about Benito Pérez Galdós Avenue and Catedrático Ferre Vidiella Street. The building has 69 homes with two, three, and four bedrooms, as well as parking spaces and storage rooms. It has eight different types of dwellings (A-F, Penthouse N-P). It is a building between dividing walls, in the corner, ground floor plus six and attic, with five vertical communication cores: four of them with access from Benito Pérez Galdós Avenue, and the fifth one with access through Catedrático Ferre Vidiella Street.

**Fig 2:**
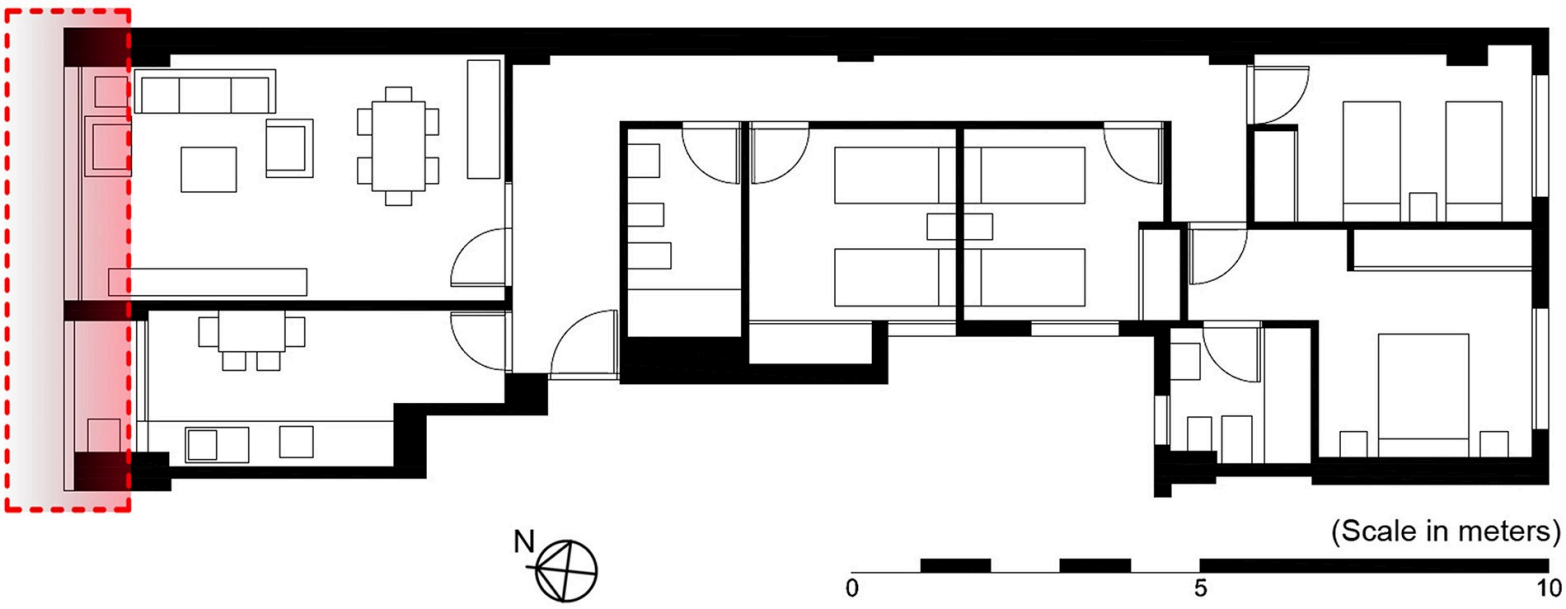
Floor plan of the type C dwelling. The red area represents the monitored ventilated facade.

The dwelling under study corresponds to type C of the typologies of dwellings in the building, and is located on the third floor, with north and south facades. It consists of two bathrooms, four bedrooms (two bedrooms facing the inner courtyard of the building of 4 x 4 *m*^2^, and two bedrooms facing the inner courtyard of the block), and a living-dining room and kitchen facing the north facade, which adjoins the Benito Pérez Galdós Avenue (Fig 2). None of the boundaries of the dwelling correspond to the median, and therefore all its partitions adjoin dwellings of the same development.

The opaque cladding of the northwest facade is composed by the following layers and technical characteristics: ventilated facade applied on perforated brick layer, with aluminium profiles and quadrangular tubes substructure, continuous insulation layer of projected polyurethane foam of 2.25 cm thickness, and finished with pieces of porcelain stoneware tiles of 10 mm of thickness.

A thermofluxometric study of the opaque enclosure was carried out in order to obtain the actual measurement of thermal transmittance U [31], as established in standard ISO 9869–1:2014 [32]. The equipment consists of a thermal flow plate, a transducer that generates an electrical signal proportional to the total heat rate floor applied to the sensor surface. Two outdoor and indoor air temperature probes are attached to the data logger, as well as two outdoor and indoor wall surface temperature sensors. The data analysis was carried out over a week with the module for calculation of the thermal transmittance of the AMR WinControl software developed by Ahlborn for ALMEMO measuring instruments. The method used was the “average method” [33].

The calculation of the thermal transmittance U (Table 1), was also carried out, according to thicknesses measured in situ, and thermal conductivity values λ obtained in the laboratory through a C-Therm TCi thermal conductivity analyser from Mathis Instruments Ltd. with universal sensor, carried out at the University of Alicante [34]. For this purpose, according to the Technical Building Code (CTE), regulations applicable in Spain [35], a surface thermal resistance of 1/hi per 0,13 *m*^2^ C/W, a surface thermal resistance of 1/he per 0,04 *m*^2^ C/W, and a thermal resistance Rc of the ventilated facade chamber of 0,176 *m*^2^ C/W, considered this as a weakly ventilated chamber.

**Table 1:** Value of the thermal transmittance λ taken from the standard UNE EN ISO 10456:2012, which establishes the Spanish CTE.

	Vertical enclosure and horizontal flow	Thermal Resistance		
LAYERS		Thicknesses	λ	R
		*m*	*W*/*m* ⋅ *K*	*m*^2^ ⋅ *K*/*W*
1	Outdoor environment (Rse)			0.04
2	Discontinuous porcelain stoneware cladding	0.01	2.3	0.005
3	Low-ventilated air chamber	0.07		0.176
4	Projected polyurethane thermal insulation	0.0252	0.028	0.9
5	Perforated ceramic brick	0.115	0.75	0.153
6	Hollow ceramic brick	0.115	0.52	0.221
7	Plaster coating	0.015	0.27	0.056
8	Indoor environment(Rsi)			0.13
	*R*_*T*_ = ∑ *R*_*j*_(*m*^2^ * *K*/*W*)			1.681
	*U*_*value*_ = 1/*R*_*T*_(*W*/(*m*^2^ ⋅ *K*)) = 0.596			

The air conditioning system of the house consists of a VRV split inverter system, with the condensing machine located on the roof of the building, and an evaporator of 3,200 W of power located in the false ceiling of the general bathroom. The treated air is distributed through rectangular fibreglass ducts.

### 2.1 Monitoring

The enclosure of a residential building located in Alicante, Spain, on the Mediterranean coast has been monitored. This enclosure was monitored during the complete cycle of 2012 and 2013, in order to be able to properly evaluate its thermal behaviour in the different seasons, and to compare the effect of the thermal inertia of its materials and incident solar radiation.

**Fig 3:**
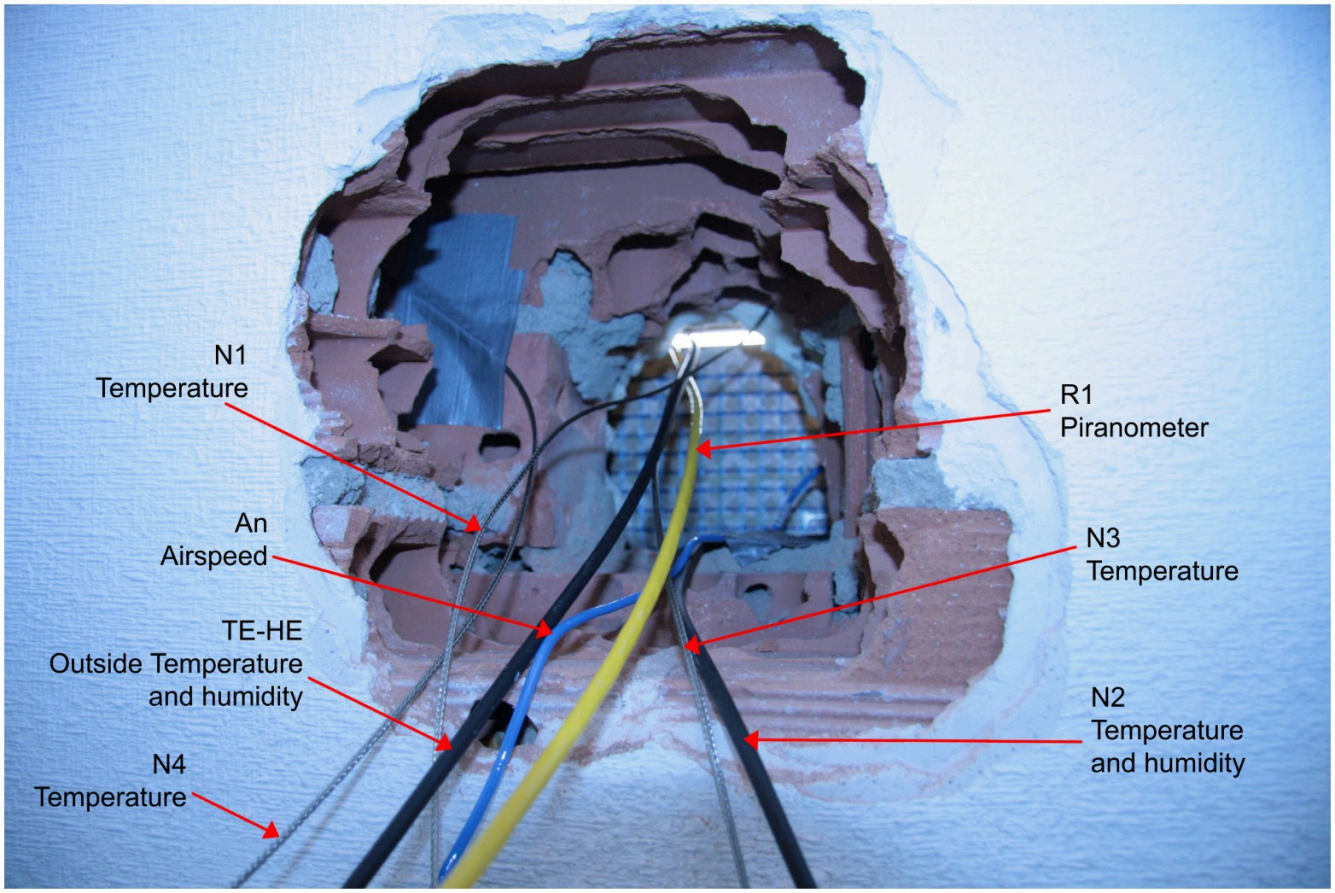
Location of sensors in the enclosure layers.

The monitoring equipment was installed a few years after the construction of the building. In order to introduce some of the surface temperature sensors, a quasi-circular surface of approximately 40 cm diameter, four layers of the facade had to be broken [36]. The hole made in the insulation layer was about 25 cm in diameter (Fig 3). This installed equipment consists of sensors for temperature and relative humidity of the outside and inside air, sensors for surface temperature in the different layers of the facade, velocity of the inside air and the outside chamber, and solar radiation by means of a pyrometer located in a facade 1m from this one.

PT 100 surface temperature sensors were also installed in all interior layers of the facade. This was done with a destructive layer system, causing a perforation of approximately 0.40 m in diameter in the bottom right-hand side. In this way, the sensors were introduced through the interior in all five layers that make up the wall.

Subsequently, these layers were closed again with materials similar to the original ones, generating some discontinuities in construction materials due to construction difficulties. The thermal gradient curves could be plotted at any time of the year, as well as their variations in dynamic regime.

The technical description of the sensors used in this study is as follows:

- GPRS RTU, 1 RS232/385 Port, two pulse inputs, 14 digital inputs, six open collector digital outputs, six 10 V analogue inputs. Modbus master.
- Standard Hygroclip2-HC2-S temperature and relative humidity probe. Temperature range of −50 to + 100°C (−40 to + 60°C, 1 V outputs), relative humidity 0% to 100%. Accuracy ± 0.8% rh, ± 0.1 K.
- Standard Hygroclip2-HC2-S3 temperature/relative humidity probe for meteorological applications. Temperature range of −50 to + 85°C, relative humidity 0% to 100%. Accuracy ± 1% rh, ± 0.3°C.

**Fig 4:**
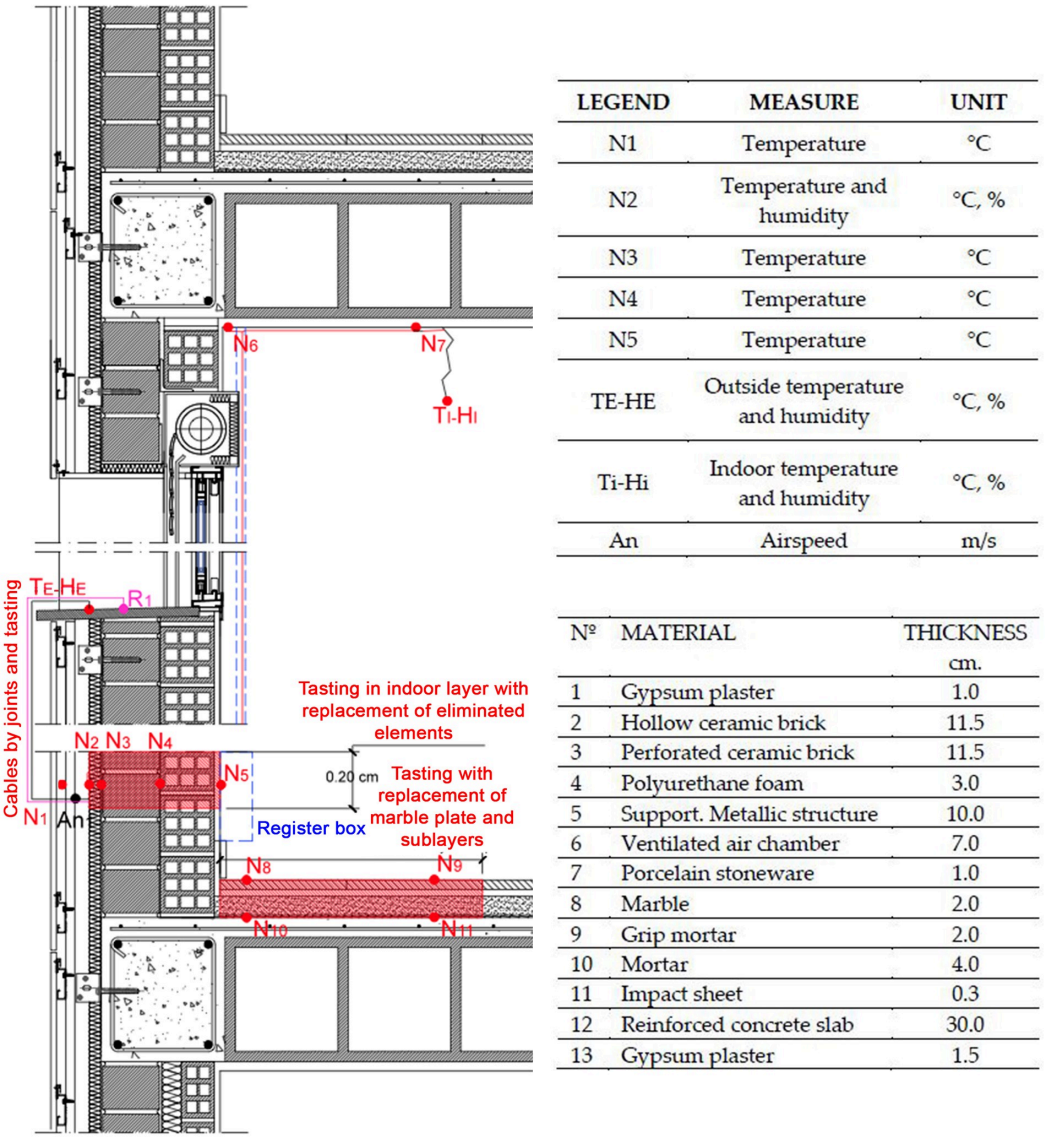
Chart of sensor location.

Fig 4 shows the position of the sensors in the facade.

## 3 Theoretical modelling

The Fourier law, is a electric analogy applied to heat transfer phenomena in walls made by parallel layers of materials of unlimited surface and stationary regimes of indoor air temperatures *Ti* and outdoor temperatures *Te*, establishes the temperature gradient that multi-layer facades acquire when there is a thermal jump in temperatures between the outdoor air and indoor air, as shown in Eq 1. It involves thermal transmission by conduction, between the various materials or layers, and convection, according to values of heat resistance or surface thermal resistance due to the convection currents generated.

$$U-value=\frac{1}{R_{T}}=\frac{1}{\frac{1}{hi}+\sum_{0}^{n}\frac{e_{i}}{\lambda_{i}}+\frac{1}{he}}$$ (1)

Where:

- *R*_*T*_, Total thermal resistance to heat transfer $\left(\frac{m^{2}{\;}^{{}^{\circ}}C}{W}\right)$
- $\frac{1}{hi}$, Interior surface thermal resistance $\left(\frac{m^{2}{\;}^{{}^{\circ}}C}{W}\right)$
- $\frac{1}{he}$, External surface thermal resistance $\left(\frac{m^{2}{\;}^{{}^{\circ}}C}{W}\right)$
- *e*_*i*_, Thickness of each layer (*m*)
- λ_*i*_, Thermal conductivity of the material in each layer $\left(\frac{W}{m^{{}^{\circ}}C}\right)$

The value of the transmittance *U* of the enclosure, which is the inverse of the value of the total resistance to heat passage *R*_*T*_, represents the value of the heat flow in watts that will occur between the outdoor environment and the indoor environment per unit of surface area (*m*^2^) and for each *°C* of temperature difference *Te*-*Ti*. This parameter is used in building design to quantify the annual energy demand, the thermal comfort of the indoor environment, the thermal loads for the design and dimensioning of the air conditioning systems, and to assess the risk of interstitial condensation, which could cause damage over time. The formulation of Fourier’s laws works in each layer in the classic linear approach only if material is solid and fixed (this is not the case of air in enclosures). The Fourier law, is a electric analogy applied to heat transfer phenomena in walls made by parallel layers of materials of unlimited surface and stationary regimes of indoor air temperatures. The walls have limited dimensions, discontinuities due to carpentry and glazing, thermal bridges due to construction requirements [11], some permeability to air passage, and the risk of interstitial condensation. The various layers that make up a wall are often made up of materials of different natures, with physical parameters of heat resistance, diffusivity *α*, effusiveness *ρ* and calorific capacity *Cp* of various values [37]. Diffusivity is the parameter that allows the heating rate of the different layers of the facade to be determined. At a high diffusivity, the material transmits heat more quickly, corresponding to a high rate of thermal transmission:

- *α* $=\frac{\lambda }{\rho \cdot Ce}$
- *α*, Thermal diffusivity $\frac{m^{2}}{s}$
- λ, Thermal conductivity $\left(\frac{W}{m^{{}^{\circ}}C}\right)$
- *ρ*, Density $\left(\frac{Kg}{m^{3}}\right)$
- *Ce*, Specific heat $\left(\frac{J}{Kg{\;}^{{}^{\circ}}C}\right)$

Thermal effusivity is the parameter that determines the capacity of materials to accumulate heat energy, equivalent to the concept of thermal inertia. The higher the thermal conductivity, density, and specific heat of a material, the greater the diffusivity and heat storage capacity.

- $\epsilon =\sqrt{\lambda \cdot \rho \cdot Ce}$
- *ϵ*, Thermal effusivity $\left(\frac{s^{\frac{1}{2\cdot }}W}{m^{2}{\;}^{{}^{\circ}}C}\right)$
- λ, Thermal conductivity $\left(\frac{W}{m^{{}^{\circ}}C}\right)$
- *ρ*, Density $\left(\frac{Kg}{m^{3}}\right)$
- *Ce*, Specific heat $\left(\frac{J}{Kg{\;}^{{}^{\circ}}C}\right)$

The thermal inertia of the materials that make up the enclosure, based on thermal conductivity λ, the specific heat *Ce* and its density *ρ*, alters the linear Fourier process depending on the time, producing a phase shift df and a damping of the thermal wave. The phase shift of the thermal wave is the time it takes for energy to pass through the construction element:

- $df=0,0167.\frac{t}{2}.\sqrt{\frac{\rho \cdot Ce}{\pi .\lambda .t}}.d$
- *df*, Disruption of the thermal wave, usually varies by hours.
- λ, Thermal conductivity $\left(\frac{W}{m^{{}^{\circ}}C}\right)$
- *ρ*, Density $\left(\frac{Kg}{m^{3}}\right)$
- *Ce*, Specific heat $\left(\frac{J}{Kg{\;}^{{}^{\circ}}C}\right)$
- *T*, Period of time of the phenomenon. In climatic applications it is 24 hours.

Thermal wave damping is the percentage of energy that had begun to pass through the construction element, but that is not capable of reaching the interior environment of the room:

- $fa=1-e^{\left(-0,0167.\frac{t}{2}.\sqrt{\frac{\rho \cdot Ce}{\pi .\lambda .t}}.d\right)}$
- *fa*, Thermal wave cushioning.
- λ, Thermal conductivity $\left(\frac{W}{m^{{}^{\circ}}C}\right)$
- *ρ*, Density $\left(\frac{Kg}{m^{3}}\right)$
- *Ce*, Specific heat $\left(\frac{J}{Kg{\;}^{{}^{\circ}}C}\right)$
- *T*, Period of time of the phenomenon. In climatic applications it is 24 hours.

**Fig 5:**
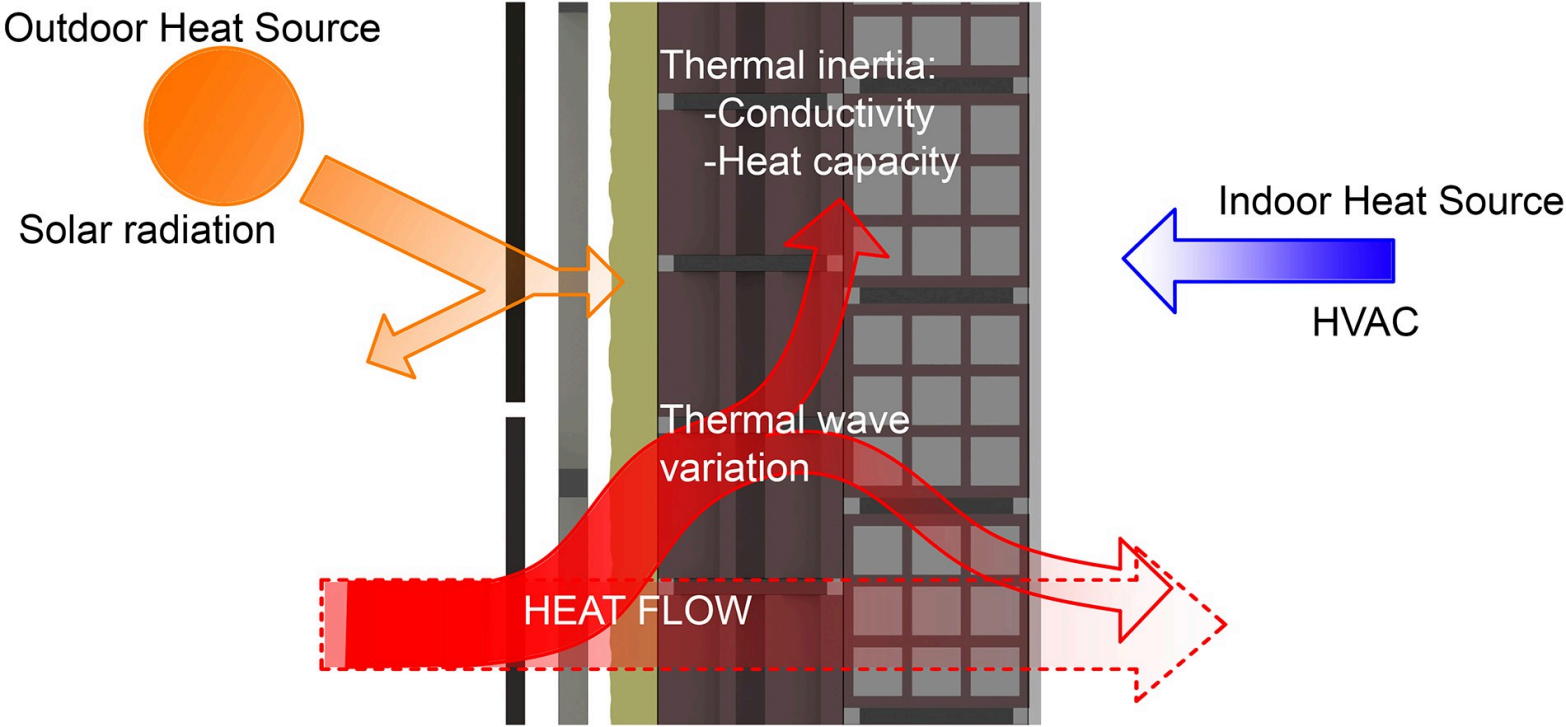
Diagram of the dynamic process of heat transmission through the enclosures.

Part of the heat flow is stored in the enclosure materials, or given up if the direction of the night phase flow is reversed. Finally, solar radiation also frequently affects the exterior surfaces substantially increasing their temperature, and therefore the flow of heat into the indoor environment. In the case of the indoor environment, the various internal thermal loads, as well as the air conditioning systems, alter the physical parameters of temperature and relative humidity of the indoor air, and the surface temperatures of the walls [38]. The heat flow thus becomes a dynamic and complex process [39] (Fig 5).

Once the theoretical model had been presented, it was decided to evaluate its functioning by comparing it with the actual measurements obtained. Specifically, once the filtering corresponding to the data had been carried out, there were 23,052 valid readings. A reading was made every 30 minutes, and an approximate time interval of 480 days was covered, obtaining 240 readings per day (48 for each of the five sensors arranged in the facade).

Several metrics have been used to compare actual sensor readings of *Y*_*i*_ to the prediction made by the theoretical model $\hat{Y}$. This comparison has been carried out independently for each insulation of the enclosure. The metrics used were the Mean-Square Error (MSE, see Eq 2) of all data and the MSE of a typical validation set with a representative sample of 20% of the total data set. This measure was then be used to facilitate comparison with the neural network model.

$$\mathrm{MSE}=\frac{1}{n}\sum_{i=1}^{n}{\left(Y_{i}-\hat{Y_{i}}\right)}^{2}$$ (2)

The mean absolute error has also been used (see Eq 3), to compare the average absolute difference between the prediction and the model. Finally, the mean and standard deviation of the difference in predictions are also attached ($Y-\hat{Y}$).

$$\text{MAE}=\frac{\sum_{i=1}^{n}|Y_{i}-{\hat{Y}}_{i}|}{n}$$ (3)

With this analysis it can be easily observed that the average absolute error of the Fourier prediction is 1.8°C, being therefore of little utility as a predictive model of the error obtained in the Eq 1, because of the static character of the Fourier prediction.

**Fig 6:**
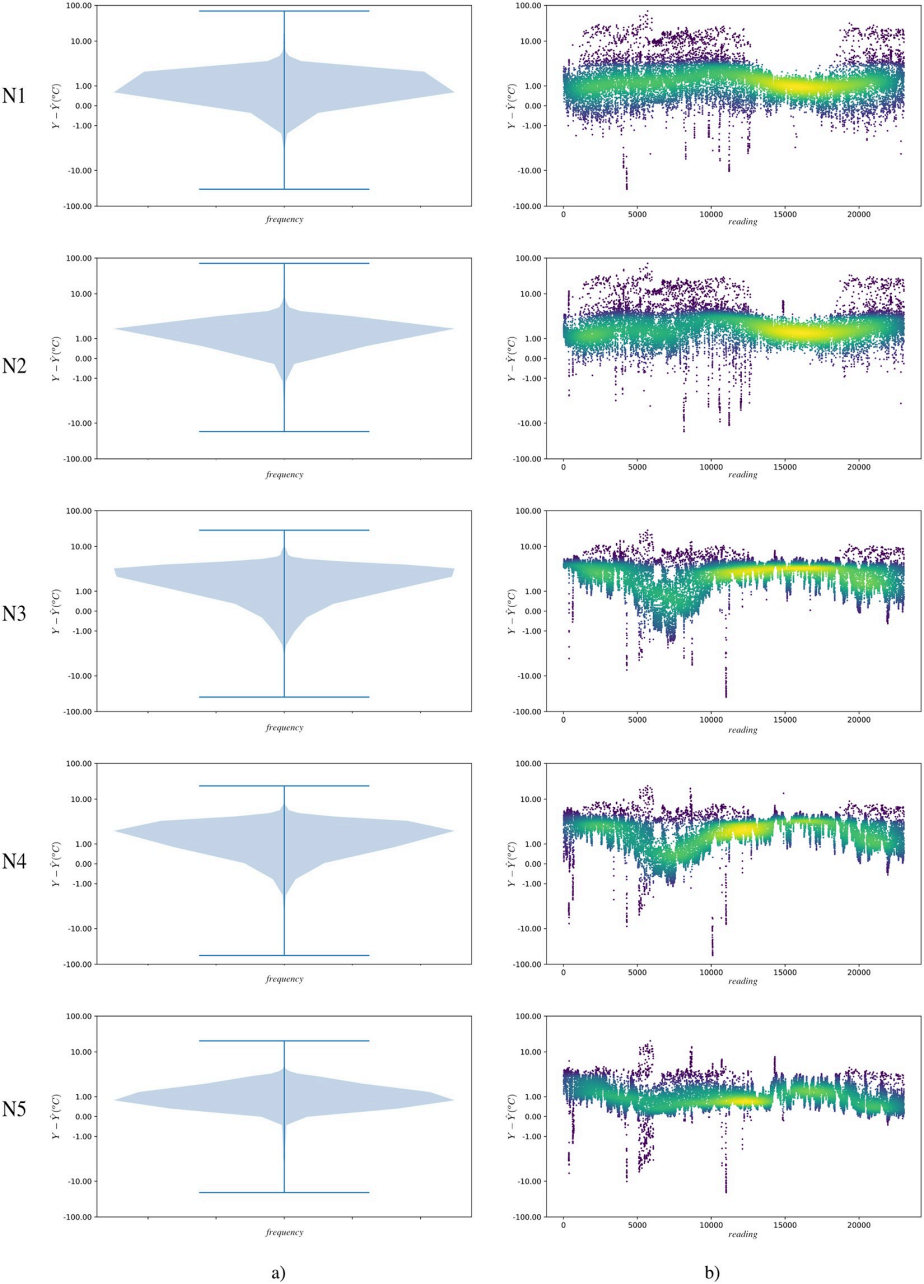
a) Violin graphics to represent the difference of actual data from the theoretical Fourier model ($Y-\hat{Y}$) for each isolation. This graph allows the easy visualisation of the distribution of the data and its probability density. b) Direct representation of ($Y-\hat{Y}$). Colour represents the density of points in a given area. Lighter colour (yellow) determines higher data density. It should be noted that both graphs are presented on a logarithmic scale. The differences between predictions are shown in Celsius degrees.

In order to be able to visualise in more detail the predictive quality of the theoretical model, the difference in the prediction of the model with respect to the real data ($Y-\hat{Y}$) is presented (see Table 2). In Fig 6, the prediction difference is presented for each of the isolations, as well as the violin graph of said difference, where it is possible to observe the density of the prediction error. It is observed that the model does not behave in a stable way in terms of prediction with respect to the actual data obtained.

**Table 2:** Comparison of actual reading data *Y* compared to predictions $\hat{Y}$ by applying the Fourier theoretical model. Mean-square error and mean absolute error are presented for all readings as well as for a test set containing a random 20% sample of the data. The mean and standard deviation of the difference in predictions are also attached ($Y-\hat{Y}$). The differences between predictions are shown in Celsius degrees.

Layer	MSE	MSE Test.	MAE	MAE Test.	$\mu (Y-\hat{Y})$	$\sigma (Y-\hat{Y})$
N1	10.2654	10.3203	1.6231	1.6263	1.4928	2.8349
N2	13.9785	13.7576	2.1095	2.0770	2.0444	3.1303
N3	7.9670	8.3208	2.3064	2.3052	2.1886	1.7824
N4	6.2510	7.2774	1.8905	1.9272	1.7463	1.7893
N5	2.0348	2.0821	1.1135	1.1317	1.0315	0.9853

## 4 Deep Learning Modeling

### 4.1 Network design

As mentioned above, a model is required that is capable of predicting the temperature of different areas of a facade. As input data both the outside temperature *Te* and the inside temperature *Ti* of the facade are used. There also exists a history of readings of the different temperatures, both internal and external to the facade. Multiple readings have been taken over a period of about one-and-a-half years at 30-minute intervals.

Although the theoretical model presented above does not take into account previous temperature data, it seems reasonable to contemplate the “memory” effect of the insulation materials and therefore to include a limited number of previous values in the model. It has also been considered that the distinctive characteristics of each insulation type used in the facade require specific modelling.

These characteristics guide the proposal of the use of Machine learning. Machine learning is a form of data analysis that automates analytical model building. The field of construction is well placed to benefit from the advent of machine learning and artificial intelligence. Deep Learning is an advanced new approach to Machine Learning, where the algorithm defines an end-to-end computation: from the raw sensor data all the way to the final output. In this model, the algorithm must figure out for itself what the correct features are and how to compute them. This results in a much deeper level of computation.

More specifically, a neuronal model is able to draw conclusions from temperature readings and a certain enclosure that includes special features (imperfections, environment, reading errors …), impossible to predict and describe in a general theoretical model. These algorithms work better the more examples they have for its training. As we will see, this is why we have determined that a whole year of training data will be provided to the model to take into account an entire seasonal cycle.

We will use feed-forward time-delay neural networks (TDNN) for this model. These networks are a promising and potentially high-potential method for forecasting time series [40] and other time-dependent problems [41].

#### 4.1.1 Temporary forecast using TDNN

The prediction of time series in the field of neural networks can be done in several ways. There are two different lines that allow the requirements for this type of series to be established. On the one hand, time can be represented explicitly, with recurrent connections of the output nodes to previous layers. On the other hand, an implicit representation of time can be established by providing a Multilayer Perceptron (MLP) network with dynamic properties.

Recurrent Neural Networks (RNN) assume that the sequence of the data has a temporal relationship. The memory feature of the RNN structures can capture this temporal information by learning time dependencies. The RNN networks are capable of handling sequences of different sizes, up to a certain maximum value. In addition, these types of networks are beneficial if the most important data points are always at the end of the sequence.

In our case, it seems reasonable that the time memory required by this problem is limited. We need a small and fixed sequence size, where each element of the sequence has the same importance. We assume that the temporal sequence is neither long nor complex since the influence of the external agents to the enclosure is diluted with time. One simple way of building short-term memory into the structure of a neural network is through the use of time delay, which can be implemented in the input layer of the neural network. A network can be made dynamic by providing it with long-term or short-term memory terms, in the structure of a classical neural network. An example of such architecture is a time-delay neural network, which is used in this article.

TDNNs is a simple way to represent mapping between past and present values. The delays in the TDNN remain constant throughout the training procedure and are known before training. In this way, we consider that this type of model is appropriate for the required prediction task. Moreover, there are several studies that validate that both types of networks work in an equivalent way in the problem of temporal sequences predictions [42–44].

Time-delay neural networks, originally designed for speech recognition, performs excellently in the modelling of dynamic systems with a time delay [40], [45], [46].

The response of these neural networks in time *t* is based on the inputs in times (*t* − 1), (*t* − 2), …, (*t* − *n*). In this case, a mapping performed by the TDNN *f* produces a *y*(*k*) output at time *k* as:

y(k)=f(u(k),u(k−1),…,u(k−M))

where *u*(*k*) is the input at time *k* and *M* is the maximum adopted time-delay.

After being adequately trained, TDNN have been used successfully for prediction, because they are able to capture the dynamics of a system and to foresee the output at the current time.

#### 4.1.2 Model design

**Fig 7:**
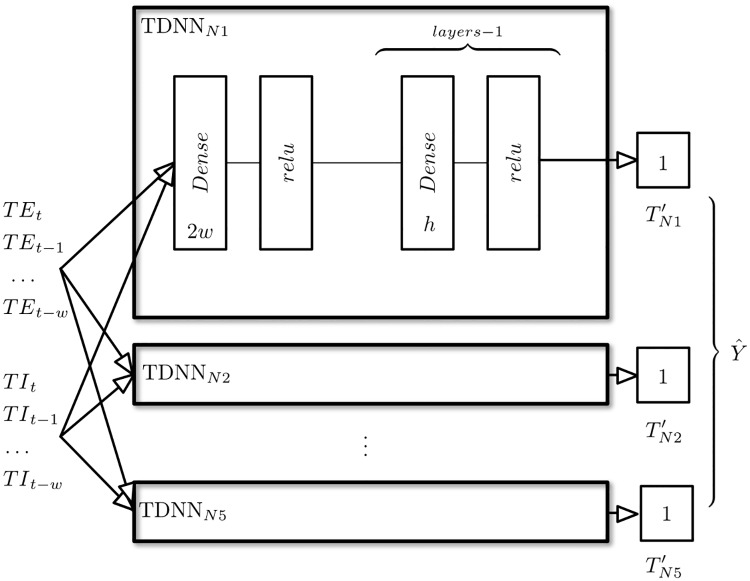
TDNN network architecture used. Five nets were used to model each of the insulation types that form part of the facade. Each TDNN network will be characterised by a specific value *w*, *h*, adn *layers*.

The TDNN’s network architecture used to model the enclosure is presented in Fig 7:

As can be seen, each insulation layer will have its own TDNN network, which will be in charge of making the temperature prediction, given the *w* of previous indoor and outdoor temperature data. The model presented requires determining the number of layers *layer*, the number of neurons per layer *h* and the number of temperatures prior to use *w* (although due to the physical characteristics of this problem this number will be very limited, and in this case it will be assumed that it will not exceed one day).

The output from each of the TDNN networks will be the temperature value predicted in isolation for the next instant (30 minutes after the current state). As this is a prediction problem, MSE will be used as a cost function to optimise the network, where *θ* are the weights of the network, *n* is the number of training examples, *x*_*i*_ is the training element *i*, *y*_*i*_ the class of that element and *h*_*θ*_(*x*_*i*_) the prediction of the network given the weights *θ*.

$$J\left(\theta \right)=\frac{1}{n}\sum_{i=1}^{n}{\left(h_{\theta }\left(x_{i}\right)-y_{i}\right)}^{2}$$

For the network training process, back propagation with the optimiser Adam is used, with the parameters (*lr*, *β*_1_ = 0.9, *β*_2_ = 0.999, *ϵ* = 1*e* − 08, *lr*_*decay*_).

### 4.2 Training process

#### 4.2.1 Hyperparameter determination

To optimise the parameters discussed above (*lr*, *lr*_*decay*_ *layer*, *h*, *w*) a randomised search was performed, as specified in [47] verifying its impact on the model by cross-validation. Table 3 shows the hyperparameters for each of the TDNN networks obtained for each insulation type.

**Table 3:** Hyperparameters obtained for each of the insulating elements of the enclosure. It shows the learning rate, the decay rate, the number of layers, the number of neurons per layer and the amount of historical data used (data corresponds to one reading every 30 minutes). In addition, the cost of training and testing is provided for each layer.

Layer	*lr*	*lr*_*decay*_	layers	h	w	Loss	Test Loss
N1	5.090E–04	2.040E–06	13	98	39	0.3026	0.2224
N2	6,536E–04	4.710E–06	8	150	46	0.6337	0.4853
N3	4.534E–04	3.767E–06	7	146	36	1.3835	0.8599
N4	1.001E–04	1.255E–06	7	126	45	1.3835	0.8599
N5	8.982E–04	4.454E–06	6	113	40	0.4272	0.2818

#### 4.2.2 Training

In this paper, the data series is divided into training and test sets. The training set is used for parameter estimation as well as to measure network generalisation. Of the data available in the training set, 20% is used as a validation set to determine the hyperparameters of the model. The test set is used exclusively to present the final performance results. To automate testing, early-stopping is used, where the training is stopped when the mean absolute error (MAE) increases in order to obtain a network having good generalisation performance. Table 3 shows the training and test error rate for each TDNN model. In addition, Table 4 includes, among other metrics, the MAE and MSE of the validation set for each enclosure insulator.

**Table 4:** Comparison of actual data *Y* compared to prediction $\hat{Y}$ made using the TDNN model. Mean-square error and mean absolute error are presented for all readings, as well as for a test set containing a random 20% sample of the data. The mean and standard deviation of the difference in predictions are also included ($Y-\hat{Y}$). The differences between predictions are shown in Celsius degrees.

Layer	MSE	MSE Test.	MAE	MAE Test.	*μ*	*σ*
N1	0.2512	0.2161	0.3089	0.3018	0.0415	0.4995
N2	0.5457	0.6336	0.4561	0.4532	0.2096	0.7084
N3	0.9873	1.1134	0.3772	0.3939	−0.1900	0.9753
N4	0.2309	0.2458	0.2868	0.2920	−0.1608	0.4528
N5	0.3211	0.3382	0.2521	0.2477	0.1433	0.5482

### 4.3 Model results

Once the prediction model was developed, its performance was evaluated by comparing it with actual measurements. As in the theoretical model analysis, MSE and MAE metrics from actual temperature data were used with respect to the data predicted by the model. For easy comparison with the cross-section in section 3, these metrics have been applied to both the data set and the test set used.

**Fig 8:**
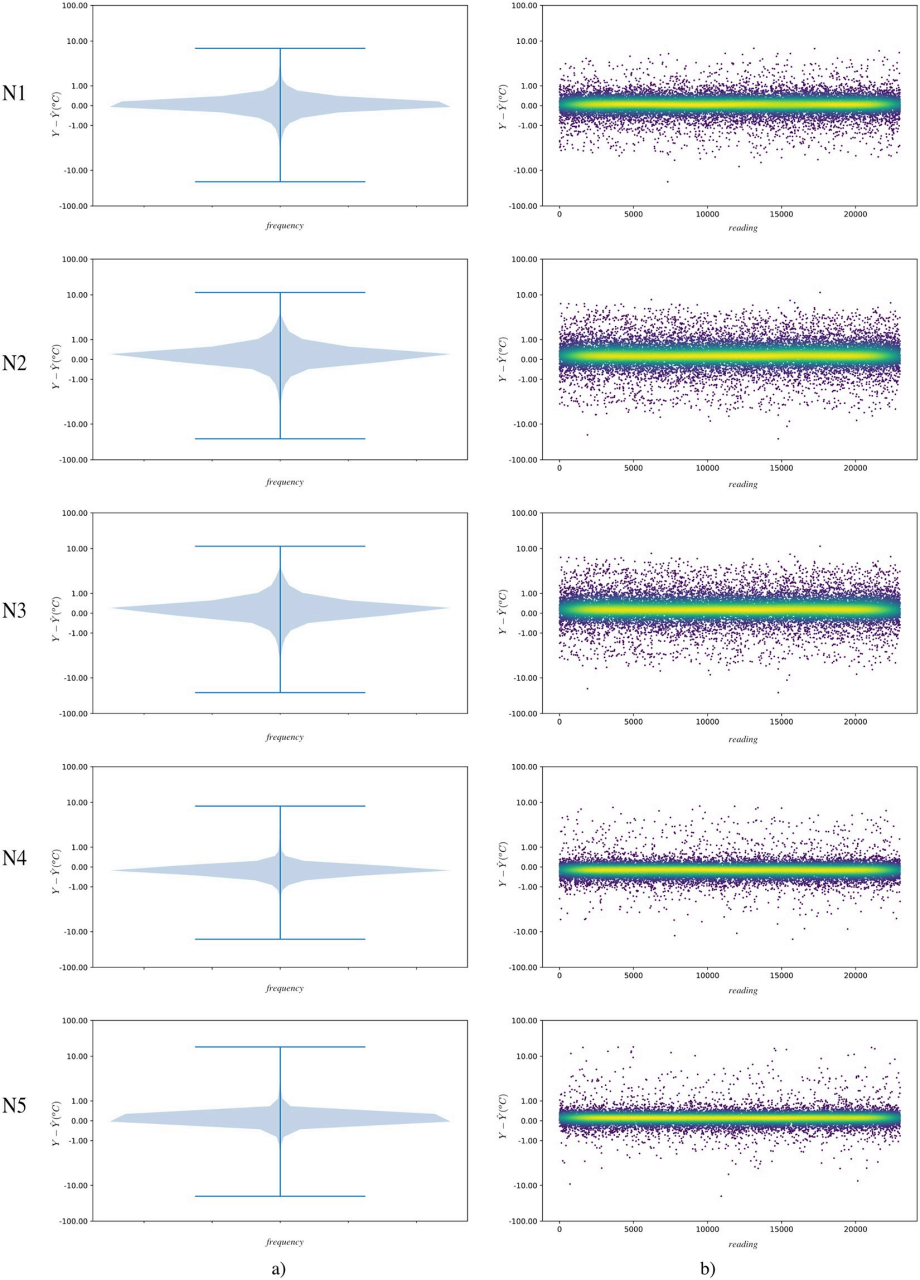
a) Violin graphics to represent the difference of actual data from TDNN model ($Y-\hat{Y}$) for every layer. This graph allows the distribution of the data and its probability density to be easily visualised. b) Direct representation of ($Y-\hat{Y}$). Colour represents the density of points in a given area. Lighter colour (yellow) determines higher data density. It should be noted that both graphs are presented on a logarithmic scale. The differences between predictions are shown in Celsius degrees.

In addition, in order to visualise the predictive quality of the model in an effective way, the difference between the prediction of the model and the real data are presented. In Fig 8 the prediction difference, as well as the violin graph of said difference, is presented for each of the isolations where it is possible to observe the density of the prediction error. The mean absolute error of the neuronal model prediction is 0.33°C for both the test set and the entire dataset.

It is important to note that after a training process that lasts no more than 1 hour (using an Intel i7 3.4Ghz CPU with a Nvidia 970GTX GPU), the network is able to make predictions in real time, with negligible response times. This is an advantage of this type of networks, compared with other models that require much more time or computing power to obtain new predictions.

## 5 Discussion

After reviewing the previous data, it can be observed how the connexionist model is able to substantially improve the prediction made by means of the Fourier theoretical model. Fig 8, shows a much more stable prediction than that found in Fig 6. In addition, the mean absolute error MAE of the neuronal model prediction is 0.33°C for both the test set and the entire dataset. Therefore, the temperature values obtained by the connexionist model are much closer to the actual readings than the average MAE error obtained by the Fourier model (1.8°C).

**Fig 9:**
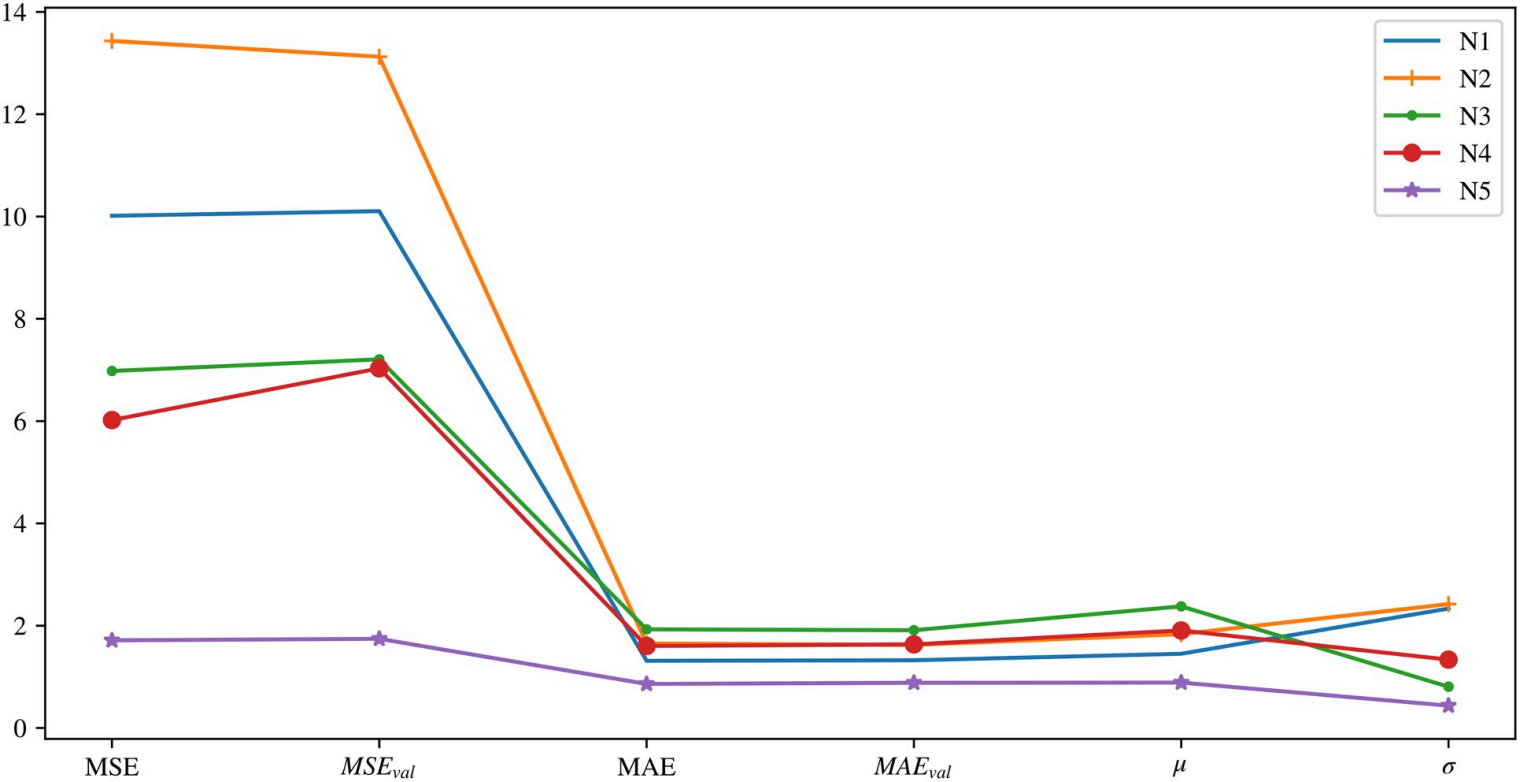
Differences in prediction metrics presented in section 3 for the Fourier model with respect to the TDNN model for each of the layers. The differences between predictions are shown in Celsius degrees.

To assess the models in more detail and establish a comparison between them, Fig 9 is shown. In this case, differences in the prediction error metrics of both models are observed. This shows how, for all the metrics used, this difference is never negative, which means that the TDNN model always improves the theoretical model.

**Fig 10:**
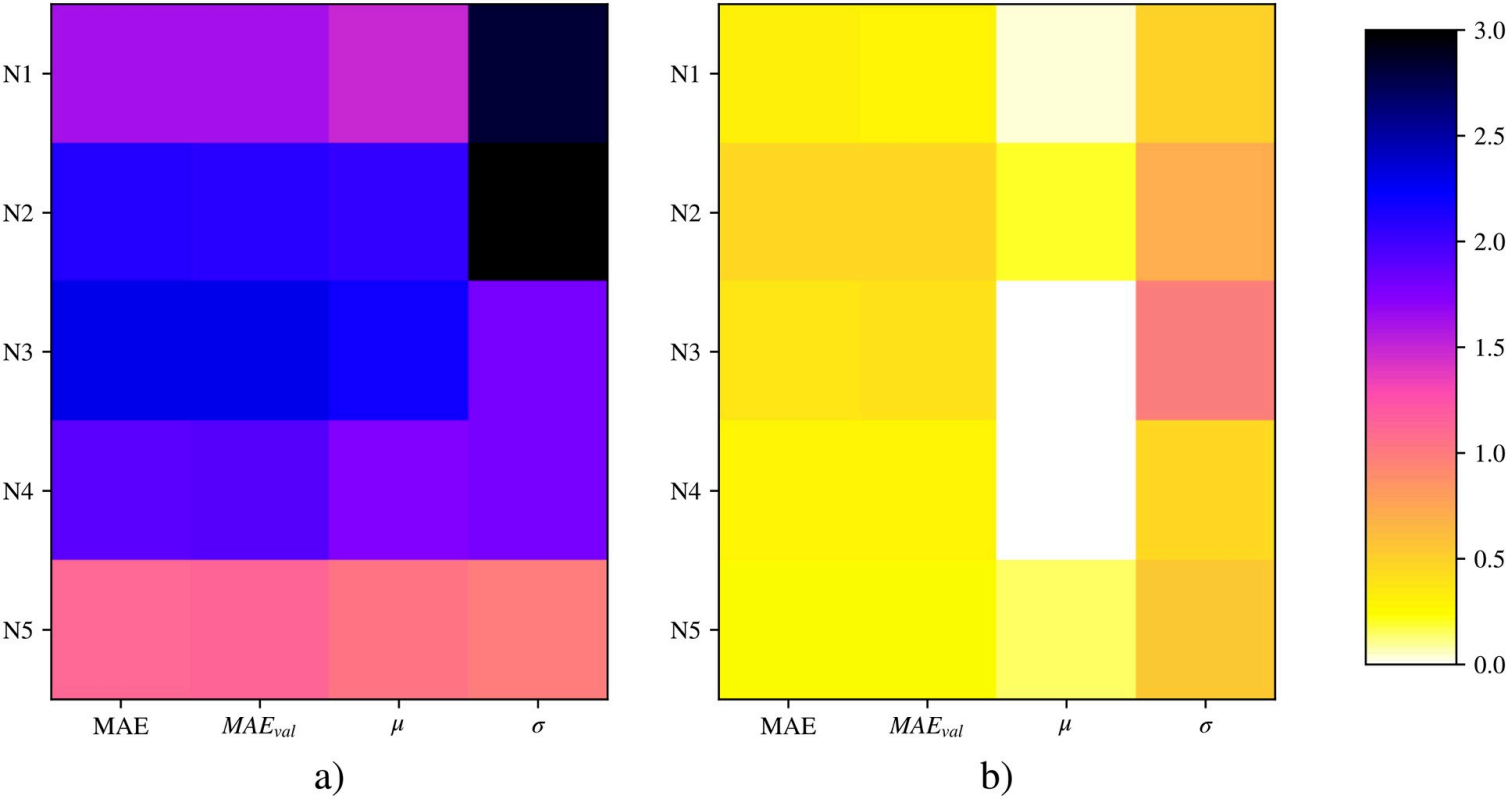
Heat graph for each layer of metrics (MAE, MAE on the test set, mean, and standard deviation) applied to the difference in prediction of actual data with respect to the model ($Y-\hat{Y}$) for: a) Fourier model, b) TDNN model. The differences between predictions are shown in Celsius degrees.

It also presents the Fig 10, which compares by means of a heat graph the MAE, MAE metrics of the test set and the mean and standard deviation of its prediction with respect to the actual data for each model.

In this case, the stability of the neuronal model is evident, where its mean error is close to zero for all isolations. The same is true for the standard deviation of differences, much less so in the connexionist case. These relationships are displayed quantitatively in Fig 9.

It is important to point out that the above graphs show a different behaviour for each of the layers of material that form part of the enclosure. In Fig 10, based on the MAE metric, it can be determined that the most complicated layers of modelling in both models are N2 and N3, followed by N4 and N1. The simplest layer is N5. This is because the N2 and N3 sensors pick up temperature variations on both sides of the thermal insulation, where the greatest thermal jumps occur, and the incidence of thermal inertia is greater when the daily oscillation of the outdoor air temperature is wide. The differences with respect to the measurements, according to the stationary or Fourier model, are thus greater. The N5 layer is the easiest to model due to the low daily variation in indoor air temperature. The internal thermal admittance Y11—the relationship between the heat flow through the exterior face of the facade and the oscillation caused by the temperature of the indoor air—is conditioned by the thermal inertia of the layers. The effect of solar radiation mainly affects the N1 layer, causing differences compared to the Fourier model, which only takes into account the surface thermal resistance 1/*he*.

The model provided substantially improves upon the stationary or Fourier prediction model. It takes into account the effects due to the effusivity and diffusivity of the materials that make up the enclosure in dynamic mode, with the daily oscillations of the outside air temperature, the effect of the heating of the outer layer due to the incidence of solar radiation, the oscillation of the indoor air temperature and the surface temperature due to the air conditioning systems, and the infiltration of air experienced by the enclosures when the pressure or temperature increases.

The design of a mathematical model for predicting the behaviour of building envelopes is extremely complex, and could not take into account aspects as relevant as the quality of construction, with the usual defects in the execution of the works, real discontinuities in materials, or permeability to the passage of air. The modelling using deep learning techniques is able to predict the actual thermal behaviour of the facade with much greater precision and simplicity. It is a highly useful model for simulating the dynamic thermal behaviour of building envelopes, and thus evaluate energy losses through the facade and the risk of interstitial condensation, which the Fourier model cannot solve.

In this way, the TDNN model presented in this research will make it possible to optimise the layout of the different layers of a facade during the design phase, the choice of materials and their thicknesses—especially the insulation layer—as well as the quantification of the building’s annual energy demand by integrating the model into simulation tools such as TRNSYS or Design Builder. It will also allow the evaluation of the action of passive conditioning systems incorporated in the facade, such as ventilated chambers, greenhouse effects, Trombe walls, or the disposal of PCM phase change materials integrated in the facade. These lines of work will be undertaken in future research.

## Supporting information

S1 File Temperature data. Temperature data obtained in the building using the sensors presented in the paper. This dataset contains 23,052 valid readings. A reading was made every 30 minutes, and an approximate time interval of 480 days was covered, obtaining 240 readings per day (48 for each of the five sensors arranged in the facade). The dataset is saved within a hd5f file for all the sensor data: Outside temperature, N1, N2, N3, N4, N5 and Inside temperature.
(H5PY)
